# Ultrasonic Influence on Plasmonic Effects Exhibited by Photoactive Bimetallic Au-Pt Nanoparticles Suspended in Ethanol

**DOI:** 10.3390/ma12111791

**Published:** 2019-06-03

**Authors:** Eric Abraham Hurtado-Aviles, Jesús Alejandro Torres, Martín Trejo-Valdez, Christopher René Torres-SanMiguel, Isaela Villalpando, Carlos Torres-Torres

**Affiliations:** 1 Sección de Estudios de Posgrado e Investigación, Escuela Superior de Ingeniería Mecánica y Eléctrica Unidad Zacatenco, Instituto Politécnico Nacional, Ciudad de Mexico 07738, Mexico; ericabrahamh@gmail.com (E.A.H.-A.); ctorress@ipn.mx (C.R.T.-S.M.); 2 Academia de Ciencias (Acústica), Escuela de Laudería, Instituto Nacional de Bellas Artes y Literatura, Querétaro 76000, Mexico; jesusalejandrott@yahoo.com.mx; 3 Escuela Superior de Ingeniería Química e Industrias Extractivas, Instituto Politécnico Nacional, Ciudad de Mexico 07738, Mexico; martin.trejo@laposte.net; 4 Centro de Investigación para los Recursos Naturales, Salaices, Chihuahua 33941, Mexico; isaelav@hotmail.com

**Keywords:** plasmonic nanoparticles, nonlinear acousto-optics, nanofluids, ultrasonic sensors

## Abstract

The optical behavior exhibited by bimetallic nanoparticles was analyzed by the influence of ultrasonic and nonlinear optical waves in propagation through the samples contained in an ethanol suspension. The Au-Pt nanoparticles were prepared by a sol-gel method. Optical characterization recorded by UV-vis spectrophotometer shows two absorption peaks correlated to the synergistic effects of the bimetallic alloy. The structure and nanocrystalline nature of the samples were confirmed by Scanning Transmission Electron Microscopy with X-ray energy dispersive spectroscopy evaluations. The absorption of light associated with Surface Plasmon Resonance phenomena in the samples was modified by the dynamic influence of ultrasonic effects during the propagation of optical signals promoting nonlinear absorption and nonlinear refraction. The third-order nonlinear optical response of the nanoparticles dispersed in the ethanol-based fluid was explored by nanosecond pulses at 532 nm. The propagation of high-frequency sound waves through a nanofluid generates a destabilization in the distribution of the nanoparticles, avoiding possible agglomerations. Besides, the influence of mechanical perturbation, the container plays a major role in the resonance and attenuation effects. Ultrasound interactions together to nonlinear optical phenomena in nanofluids is a promising alternative field for a wide of applications for modulating quantum signals, sensors and acousto-optic devices.

## 1. Introduction

The nonlinear optical (NLO) properties of nanomaterials are attractive, due to the possibility of their use in different technological fields [1]. In recent years, a great diversity of nanostructure thin films [2] has been reported observing different NLO properties which are attributable to the quantum confinement [3]. In particular, metal nanoparticles (NPs) have attracted great interest specially when are suspended in different based fluids [4], due to their physical and chemical characteristics sensitive to the local environment [5]. Also, nanofluids appear to be suitable for biomedical applications as molecular diagnostics, delivering sensing and bioimaging [6]. 

Diverse colloidal suspensions of metallic NPs have been the cornerstone of significant studies devoted to photonic [7], opto-electronic [1], electro-optical [8], and all-optical functions [9]. Multimetallic NPs display a wide range of interesting and potentially improved characteristics in comparison to monometallic NPs [10]. The superior features exhibited by multimetallic NPs are attributable to the Surface Plasmon Resonance (SPR) provided by their different components in a nanostructured configuration [11]. For example, bimetallic Gold and Platinum NPs (Au-Pt NPs) exhibit two peaks at the linear regime, which make them attractive for suitable catalyst [12] and electrochemical sensors. The formation of bimetallic alloys, such as core-shell and clusters, depends on the structure and size of the core [13], thus, they are widely influenced by the enhancement of the catalytic activity related to SPR effects [14]. Au-Pt NPs have been synthesized by different techniques as simple electrodeposition [15] and environmentally friendly procedures [16]; besides, it has been revealed the possibility to enhance their characteristics by tuning the molar ratios [17]. It must be noticed that the processing route for the preparation of the NPs plays critical roles in the size regime, morphology and distribution in comparison to the monometallic NPs [18]. Therefore, Au-Pt NPs emerge as highly promising materials for detection devices [19], and for biosensor signal processing owing to their improved physical, optical, and electromechanical properties [20]. 

The NLO response of thin films and NPs suspensions has been measured by different multiphoton absorption techniques [21]. Particularly, the NLO effects seem to be enhanced by tuning the optical field [22], which has been experimentally demonstrated in a wide range of nano-optic [23], acousto-optic [24], and acousto-plasmonic interactions. 

Acoustic waves, such as infrasound, sound, and ultrasound (US) can be propagated as a mechanical disturbance in the matter, being potentially useful since they enable the ability to induce and control low-dimensional materials. Acoustic manipulation of metallic NPs is also allowed, due to the displacement of the media’s molecules [25]. Acoustic vibrations, which have been used for enhanced-spectroscopy applications, currently have drawn interest from the NLO community [26]. Recent studies demonstrated the vibrations powered by sound in a liquid medium can generate autonomous motion, originated by nanorods trapped in an acoustic field [27]. Therefore, there is considerable interest from sound-light interaction, specially by mixing US and optical waves as a result of the rapid modulation and deflection of the light beams, as well as the demand for more general optical processing [28]. The interactions between US and nonlinear optical signals both open interesting opportunities for technological branches ranging from optics to photonics [29]. 

Also, multi-wave interactions promise potential for applications as acousto-optic modulation and compact photonic devices [30], as well as breast cancer diagnosis candidates for acousto-optic imaging in biomedicine [31]. This is because, while techniques that deal with the problem of image generation from diffuse photons suffer low signal-to-noise ratio, the light which has passed through a scattering medium can be easily detected and localized. Therefore, even a three dimensional localization of breast tumors and their characterizations can be obtained through ultrasonically-controlled signals. 

Particular properties derived from quantum confinement and the interaction between nanostructures has been indicated in metal nanostructures [32,33]. Some interesting aspects of plasmonic materials are their large absorption cross-sections and strongly localized electromagnetic fields [34]. Remarkably, the linear and NLO effects play an important role in the vibrational mode driven by acousto-plasmonic coherent control and result in a change of the refractive index [35]. In light-sound interactions, the modification of the optical refractive index is due to the particle’s compression and additional refraction effects can be originated by the sound wave propagation through a fluid media [36]. It has been demonstrated that plasmonic metamaterial-based sensors for US detection, present advantages derived from their strong sensitivity to the refractive index of their surroundings [37]. Besides, opto-mechanical nonlinearities exhibited by plasmonic metamaterials can be modulated by third-order NLO parameters [38]. Plasmonic nanostructures composed of metallic and dielectric media, exhibit tunable plasmon properties associated with the collective oscillations of free electrons [39]. And another important fact is the possibility to create hybrid systems by the combination of independent elements with the aim to enhance their characteristics and applications [40]. In view of all these considerations, within this work have been analyzed optical effects that can be related to US and plasmonic phenomena. We presented variable contributions from US to NLO transmittance in single-beam and TWM experiments; these results are a consequence of the synergistic potential of plasmonics and US for modulation of optical absorption and refractive index; respectively. Specifically, in this work, an attempt has been made for further investigate the transmittance of NLO signals may receive an opposite influence from multiphotonic absorption and the optical Kerr effect when the propagation of US takes place in a nonlinear sample. Some examples that illustrate the importance of modulating optical signals by ultrasonic frequencies are related to quantum plasmonic sensors assisted by NLO effects [41] and microfluids with properties governed by the incorporation of plasmonic nanostructures and acoustic signals [42]. In this direction, the main purpose of this work is to evaluate the influence of ultrasonic signals in the plasmonic response exhibited by nanocolloidal solutions. Herein is reported a study about the ultrasonically activated modification on the plasmonic and absorption effects exhibited by bimetallic gold-platinum nanoparticles. Different contributions related to mechanical phenomena and NLO processes were observed. The significance of this study comes from the clear indication of enhancement and inhibition of the optical transmittance related to plasmonic sensing. This work highlights the attractive nonlinear optical characteristics associated with the samples for developing photoactive advanced materials.

## 2. Materials and Methods

### 2.1. Sample Synthesis 

Colloidal bimetallic Au-Pt NPs were synthesized through a sol-gel technique accordingly to a previous report [43]. Briefly, the process was carried out as follows; the sol-gel TiO_2_ was obtained from Titanium i-propoxyde [Ti(OC_3_H_7_)_4_] used as a precursor with a concentration C = 0.05 mol/L, pH = 1.25, was dissolved in a water/alkoxide solution with a molar ratio 0.8. Then, standard solutions of Au and Pt precursors with an equivalent nominal metal concentration of 1000 mg/L each were used. The mixture of (Au + Pt)/Ti(OC_3_H_7_)_4_ revealed a molar ratio of 0.76% (mol/mol) in a total volume of 11.5 mL. The resulting solution was kept in darkness for a week before nucleation with the aim to extend their photo-response, improve their catalytic activity, stabilize the sample, and use it several times [44]. Finally, an ultraviolet (UV) light reactor was used in the photocatalytic processes for the preparation of the bimetallic sample.

### 2.2. Morphology Characterization

The morphology and size distribution of the sample were both studied by Transmission Electron Microscopy and High-Resolution Transmission Electron Microscopy (TEM and HRTEM, FEI Titan 80-300, JEM-ARM200CF) techniques. For the microscopic measurements, a drop of the studied suspension was placed on a carbon-coated Cu grid, it was allowed to dry overnight at room temperature prior to the optical explorations. TEM micrographs were acquired using a probe aberration-corrected microscope, recorded by a Gatan Ultrascan 1000 xP digital camera and operated at an acceleration voltage of 80–200 kV. Besides, Energy-dispersive X-ray analysis (EDX; JEOL JSM-7800F) were undertaken to clarify the chemical composition of the Au-Pt NPs.

### 2.3. Nanosecond Optical Single-Beam Transmittance

**Figure 1:**
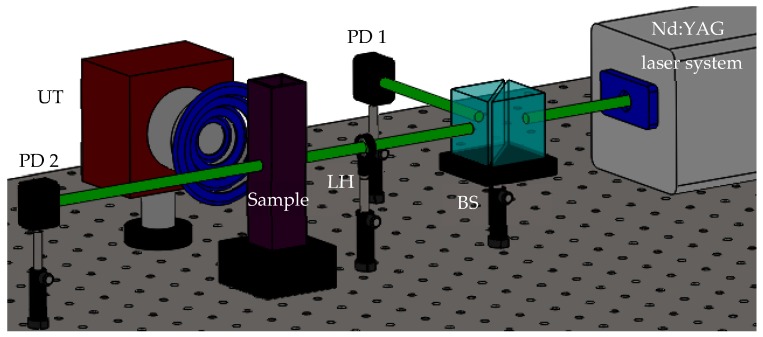
Experimental setup for single-beam transmittance measurements influenced by US.

The bimetallic Au-Pt NPs were explored by an Nd:YAG nanosecond laser source (Continuum Model SL II-10) with 4 ns pulse duration at 532 nm wavelength, repetition rate of 10 Hz, and linear polarization. Figure 1 illustrates the scheme of a single-beam experiment setup performed in our laboratory with the aim to investigate the nonlinear optical response of the solution. 

### 2.4. Acousto-Optic Explorations

In order to study the US influence on the Au-Pt NPs, the acousto-optical exploration was also investigated with the arrangement schematized in Figure 1. The excitation beam was divided by means of a beam splitter, BS, with the purpose of monitoring power emission of the laser system by a photodiode detector (PD-1) (Newport, Model: 818-BB-21). In the experiment, both light and US signals were simultaneously propagated on the colloidal sample. The optical irradiation was focused to a small spot by using a quartz lens (LH) (Newport, Plano-Convex Lens) and increased gradually while the acoustic square wave remained continuous. 

A home-built electronic circuit model by using ultrasonic transducer (UT) was employed to generate US. In accordance with the specification supplied by the manufacturer, the sensor operates in a frequency range of 40.0 ± 1.0 kHz. In order to start the operation of the UT, it must be selected a high pulse of high voltage (5V) during at least 10 μs, then, the trigger pin will transmit out 8 cycles of ultrasonic burst at 40 kHz and an ultrasonic burst is going to be reflected. The echoes from the target cannot be ignored, since they are essential to the circuit model to consider the following burst of the 40 kHz square-wave emission.

The energy of the transmitted optical beam was measured by a digital oscilloscope (Teledyne LeCroy, WaveSurfer 3054) using a photodiode detector (PD-2) (Newport, Model: 818-BB-21). Finally, both optical and ultrasonic signals were compared to evaluate the influence of the ultrasonic disturbance.

### 2.5. Two-Wave Mixing Experiment Influenced by US

**Figure 2:**
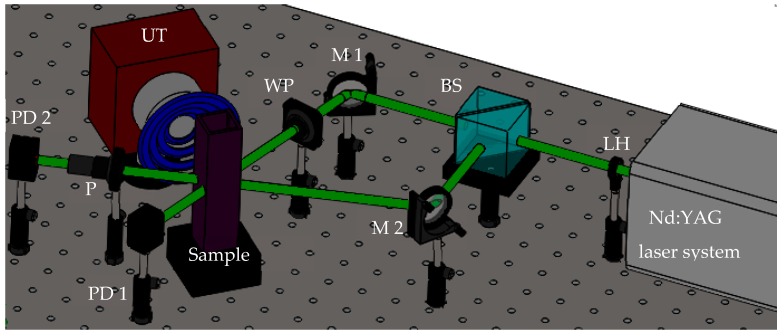
Acousto-optic explorations by the assistance of the two-wave mixing configuration.

A two-wave mixing (TWM) experiment schematized in Figure 2 was carried out to identify the vectorial nonlinear optical response of the sample. In this technique, the laser source linearly polarized with a Gaussian profile was focused by using a 75 cm focal-length lens (LH). The pulse energy of 115 mJ provided by the Nd:YAG laser system was divided into two beams by a cubic beam splitter (BS) both featuring the same light polarization direction. The two beams, pump and probe, interfere into the optical cell with an optical irradiance relation of 10:1, each beam path was guided to the sample by high-energy laser mirrors (Newport, Dual-wavelength mirror for 1064 and 532 nm. 2.0 in. Diameter), (M 1-2). An achromatic half-wave plate (WP), (Newport, Quartz-MgF2), was inserted in the pump arm to change the direction of the linear polarization with a Newport, Calcite Polarizer (P). The transmittance of the sample for both beams was measured along the laser propagation direction by PIN photodetectors (PD 1-2) (Newport, Model: 818-BB-21). The temporal and spatial superposition of the beams was verified by the optimization of the position of the mirrors considering the Kerr transmittance of the probe beam with the influence of the pump beam interacting with the sample.

For further investigate the coupling between the pump and the probe beams, we considered the right and left circular components of the electric field as $E_{\pm }$ and $E_{\mp }$. Then, we applied the finite-differences method to solve the Maxwell’s wave equation given by [45]:

(1) $$\nabla^{2}E_{\pm }=-\frac{n_{\pm }^{2}\omega^{2}}{c^{2}}E_{\pm },$$

where c is the velocity of light in the vacuum and the optical frequency is defined by ω. We assume, that both waves propagate the sample in the same plane. In our representation, is convenient to express the refractive index taking into account the approximation [45]:

(2) $$n_{\pm }^{2}=n_{0}^{2}+4\pi \left(A{\left|E_{\pm }\right|}^{2}+(A+B){\left|E_{\mp }\right|}^{2}\right),$$

here $n_{0}$ is the weak-field refractive index. $A=\chi_{1122}^{\left(3\right)}$ and $B=\chi_{1212}^{\left(3\right)}$ are the independent components of the third-order susceptibility tensor $\chi^{\left(3\right)}$.

### 2.6. Modulation of Plasmonic Signals by Ultrasonic Effects

A path length of 1 cm in a quartz cuvette was heuristically filled with the dispersion of Au-Pt NPs to explore the linear and nonlinear optical response of the samples. UV-Visible optical absorbance spectra were acquired by using an Ocean Optics UV-vis fiber optic-based spectrometer (USB 2000+XR1-ES), equipped with deuterium-halogen light source. Additionally, in order to modulate the influence of the optical response, an Ultrasonic Transducer (UT-1 and UT-2) situated above the optical quartz cuvette was used for delivering US waves in the sample. The prepared solution was scrutinized in the wavelength range from 200 nm to 900 nm.

**Figure 3:**
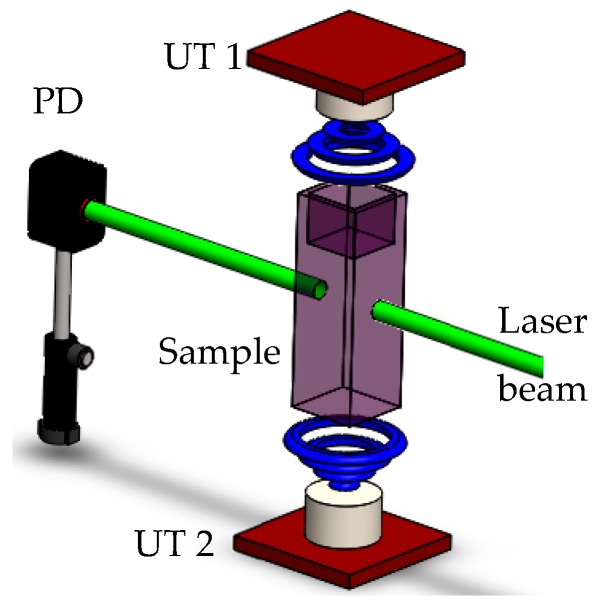
Single-beam transmittance set-up based in an Nd:YVO_4_ pulsed laser.

The modulation of the plasmonic signals was achieved by ultrasonic interactions; the experimental setup is shown in Figure 3. Near-resonance excitations were promoted by monochromatic light of λ = 532 nm provided by a horizontally polarized Nd:YVO_4_ pulsed laser (Spectra-Physics Explorer^®^ One™ XP). The laser with constant energy of 7.3 µJ was split into two beams of equal energy by means of a BS; one beam was focused on the sample using a Newport, Plano-Convex Lens with a focal length of 20 cm, while the other arm beam was used as a reference.

The sample was disturbed with US combined with the stable pulse energy of the laser source. In this case, the ultrasonic frequency was modulated by using a pair of different transducers (UT 1-2); their operation was previously described (Section 2.4). The optical quartz cuvette with the colloidal solution of Au-Pt NPs were placed between the two UT 1-2 separated at the same distance.

However, in order to obtain the phase difference frequency, we used different 40 kHz square-wave signal-each burst in 20 periods. The output light beam profile of the transmitted light was collected by a photodiode (PD) (Newport, Model: 818-BB-21) and processed by the digital oscilloscope. 

In this work, the modulation of optical signals was obtained by the combination of light with US. By using an UV-vis spectroscope is proposed to modulate the magnitude of the peak in the absorption band related to the plasmonic response of the sample interacting with ultrasonic signals. Furthermore, when the transmittance of a sample changes with the incident irradiance, then nonlinear optical effects are involved. Third-order NLO phenomena are strongly dependent on the plasmonic response of a sample; while nonlinear refraction and nonlinear absorption can be responsible for third-order nonlinear optical processes. By conducting single-beam transmittance experiments, we proposed that multi-photonic absorption processes together to ultrasonic signals is possible to increase the modulation in the transmittance of a plasmonic sample. On the contrary, due to the TWM experiments, where an induced nonlinear refractive index can be demonstrated, it is indicated the possibility to decrease the transmittance of the sample by the influence of US signals responsible for a modification in the refractive index of the sample.

## 3. Results

### 3.1. Characterization of the Synthesized Au-Pt NPs: TEM and EDX

**Figure 4:**
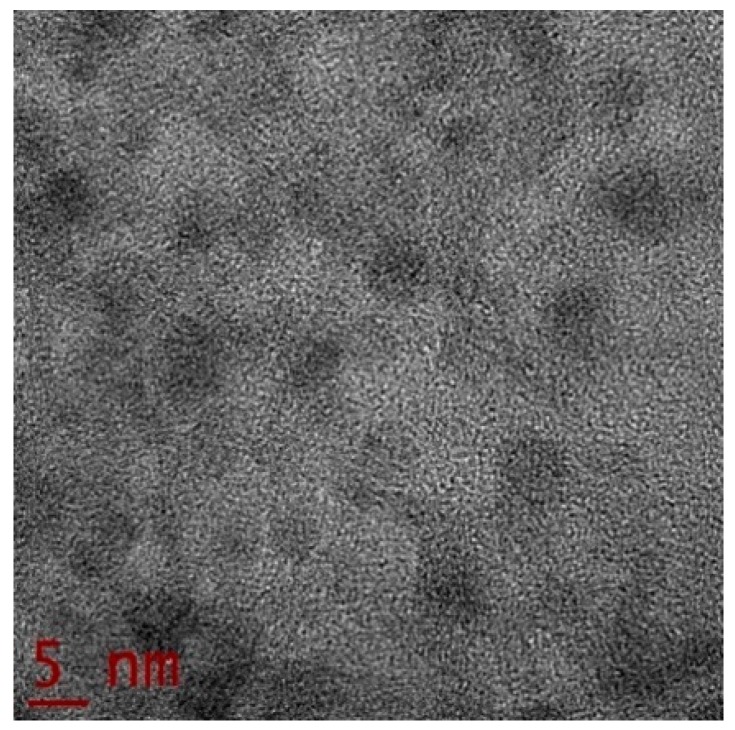
High-Resolution TEM image of the Au-Pt NPs embedded in TiO_2_ film studied.

The representative TEM image, shown in Figure 4, confirms the size and distribution of the sample. The diameter of the distributed Au-Pt NPs ranged between 9–13 nm, exhibiting an average standard derivation close to 10.5 nm. The statistics were carried out by measuring different individual particle diameters from randomly acquired TEM images. TEM can be used to perform the chemical mapping to show the Pt and Au layer on the nanoparticles. However, we guarantee the bimetallic nature in the composition of the sample by statistical EDX analysis.

**Figure 5:**
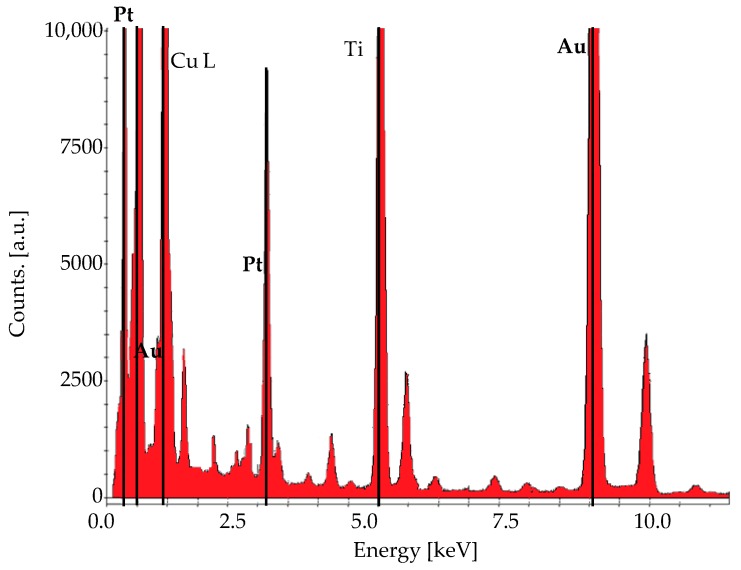
Chemical composition of the synthesized bimetallic sample studied by EDX.

The formation of Au-Pt NPs was confirmed by EDX studies mapped on Figure 5. The chemical composition of the alloy structure shows that Au is the predominant element, although the same amounts of standard solutions of Au and Pt precursors were used. The stronger photocatalytic activity exhibited by Pt seems to be responsible for this result. The atomic relation between Au and Pt in the NPs was about 10:1; respectively. EDX pattern also reveals the presence of other peaks associated from the copper substrate. We identified that increasing the Au content in Au-Pt alloys originates larger NPs which can be useful in different fields [46].

### 3.2. Single-Beam Transmittance Measurements Affected by Ultrasonic Vibrations

**Figure 6:**
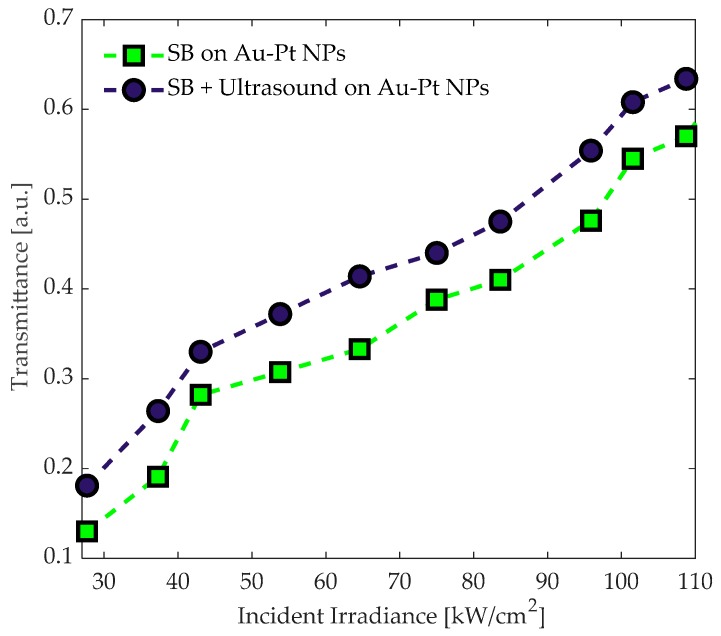
Change in the single-beam transmittance by the ultrasonic waves propagated through the Au-Pt NPs suspended in ethanol.

Acousto-optical explorations were undertaken considering that both light and acoustic waves were propagated together through the colloidal Au-Pt NPs. Figure 6 summarizes the result of the experimental setup using the Nd:YAG laser system. Initially, we measured the optical single-beam transmittance exhibited by the sample. Then, the simultaneous interaction of both optical and ultrasonic signals was considered; the blue circles’ markers depicted the final contribution of the US in the optical transmittance.

It should be noted that the interaction of both optical and ultrasonic waves influences the stability of the bimetallic colloidal solution with Au-Pt NPs. The modification of the optical transmittance as a function on US signal can be explained by considering that the US frequency is capable to modify the optical and the refractive index; since both are strongly dependent of the vibrational mode of the sample. One of the effects that would especially benefit from the contribution of the US, is that the sedimentation process decreases, due to the high-mechanical oscillations. Besides, it could prevent the appearance of agglomerations and accumulated NPs exposed to electrostatic effects. Herein, the presence of bimetallic NPs within a host liquid will not only lead to the modification of SPR effects that may improve linear and nonlinear absorption, but it could modulate their response owing to the US frequency, yielding to a larger optical absorption enhancement [47].

The morphology and particle size distribution are not affected by the precursor; however, a shift of the optical absorption spectra could be presented by decreasing the grain size [48]. Besides, solvent-metal interactions are important in NLO effects, due to the refractive index plays a prominent role in third-order optical nonlinearities [49].

In addition, the nonlinear optical properties depend on the absorption cross-section and transition energy level, as a result, the nonlinear optical transmittance increases or decreases as a function on light irradiance. In our case, the monotonic increase in transmittance, shown in Figure 6 can be considered as a signature of a typical saturable absorption dependent on incident irradiance.

### 3.3. TWM Measurements Affected by Ultrasonic Vibrations

**Figure 7:**
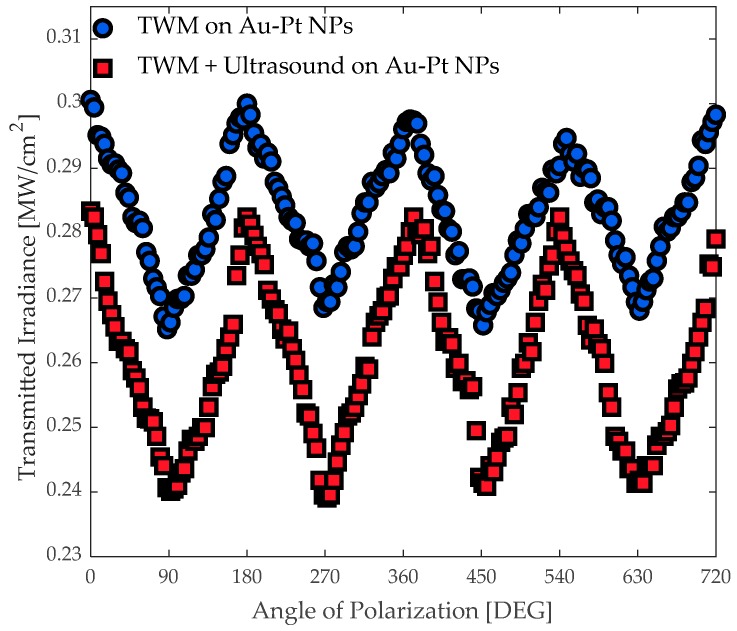
Transmitted irradiance as a function on the angle between the planes of polarization of the incident optical beams with and without an acousto-optic influence.

The nonlinear optical response of the bimetallic Au-Pt NPs suspended in an optical cell was evaluated by a nanosecond TWM technique. In this case, the angle of polarization was controlled by means of a half-wave plate (WP), (Newport, Quartz-MgF2) placed in the pump arm before to irradiate the sample. The output of probe beam provided by the Nd:YAG laser system was measured using a PIN diode PD 2 (Newport, Model: 818-BB-21). The measurements were performed for two different cases, shown in Figure 7; the blue circles represent the typical angular-dependence of the interferometric optical measurements while the plotted square red markers correspond to the values with the presence of the ultrasonic waves. The magnitude in the optical transmittance of the sample was tuned by the superposition of the waves in order to obtain an interference fringe pattern in the nonlinear medium. The ultrasonic transducer was emitting short US pulses featuring 40.0 ± 1.0 kHz square waves at a level of about 120 dB. The nanoparticle density is strongly dependent on the US frequency [50]. Therefore, the refractive index and transmittance appear to be greatly influenced by the US action. 

From the results illustrated in Figure 7, the transmitted irradiance pointed out a change in the optical nonlinearity of the sample derived by the influence of ultrasonic waves. In this case, we consider that the dynamic motion of the sample can generate an inhibition in the photoactive electronic response of the sample exposed to a near-resonance optical Kerr effect (OKE) excitation. The OKE technique has been reported to investigate the orientation dynamics of molecular liquids approaching a phase transition [51] and it has a strong dependence on the SPR properties exhibited by plasmonic NPs. The OKE manifests itself in a TWM interaction considering that a dependence on the optical irradiance can be also related to the refractive index of the media. In this TWM inside of a Kerr medium, a dependence on the host media could be remarkable because a strong influence of the US could originate changes in the density and in the induced birefringence. It is worth noting that the results plotted in Figure 7 are modulated by the angle of polarization, since the interference pattern associated with the two incident waves is dependent on the polarization of the waves. Therefore, it can be assumed that the plasmonic response of the samples is the main responsible for the modulation of the nonlinear optical transmittance controlled by the US frequency. Furthermore, it can be considered that changes in absorptive nonlinearities rising from the stationary interference pattern of the incident optical waves in the samples may contribute also to the change in the nonlinear refractive index [52]. The simultaneous participation of optical and ultrasonic interaction could be responsible for the modification of quantum confinement phenomena [11] which is related to the volume concentration, the size, and composition of the sample [18].

### 3.4. Ultrasonically-Controlled Plasmonic Signals by Bimetallic Au-Pt Nanoparticles Suspended in Ethanol

**Figure 8:**
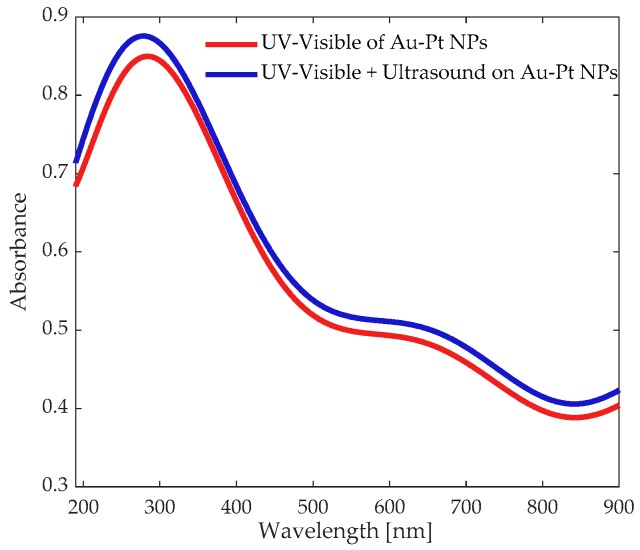
Representative UV-Visible absorption spectra of the studied samples showing a change derived by ultrasonic signals.

In Figure 8 are presented the UV-Visible absorption spectra of the sample with the participation of an ultrasonic wave excitation. These measurements represent experimental data acquired 10 different times in different regions of similar samples systematically studied. Gyroscopic behavior of the NPs seems to modulate almost uniformly the UV-vis spectra of the sample as it has been previously demonstrated [53]. In this case, the change in the linear absorption is due to a modification in the plasmonic response of the bimetallic sample. US waves are able to modulate the density in nanofluids and then, it can be expected an important modification in the collective electronic response of plasmonic samples. From the plot can be clearly seen that the colloids show a shift in the absorbance response attributed to the US wave propagation. The absorption peak presented in the UV region close to 300 nm corresponds to a plasmonic absorption of the Pt, while the absorption peak in the visible region close to 650 nm is related to the response of the Au integrating the NPs. The significance of these absorption bands is related to the selective absorptive behavior of the characteristic effects of the size, shape and distribution of each metal integrating the nanostructured sample. 

The UV-vis spectrum is associated with a random distribution of the dispersed NPs, and not only to those shown in the TEM image. Different bimetallic Au-Pt NPs alloys have been studied showing a change in the SPR peak by increasing the Au content, and due to the Au element is more electronegative than Pt [46], which originates a modification of its absorption properties and linear and nonlinear optical refraction. Hence, the possibility to change the concentration of the Au-Pt NPs exhibit unique extraordinary features induced by the plasmonic response can be attributable to the nanomechanic and nanophotonic properties of the sample.

Nanoscale effects are very important for instrumentation applications, such as plasmonic and nanophotonic sensors, regarding the high sensitivity associated with low-dimensional interactions. Also, it has been found that nanoscale phenomena are strongly dependent on the quantum confinement which appears to be improved by the participation of US frequency. The power and frequency of the US seems to be responsible for the modification of the plasmonic effects related to nanosystems [54].

**Figure 9:**
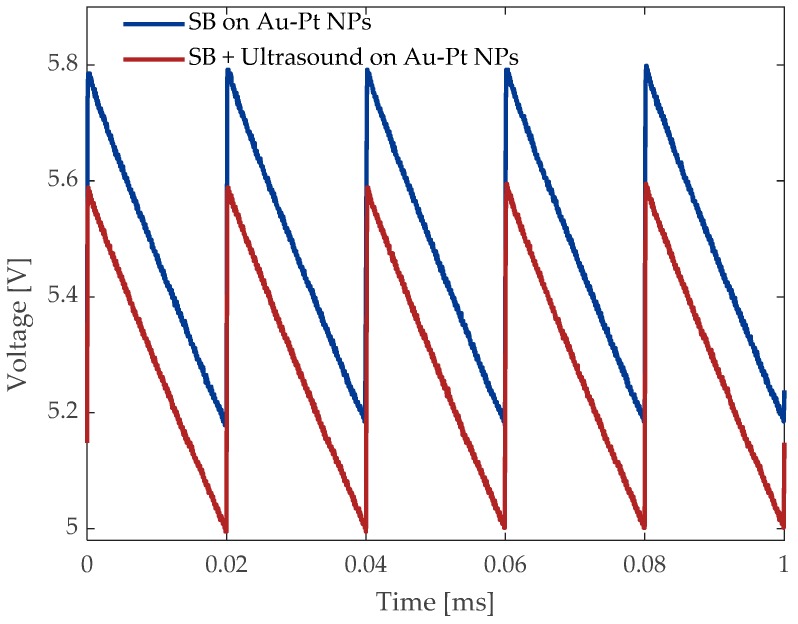
Amplitude vs time spectrum of single-beam transmittance measured at the output light with a pulse energy of 5.5 µJ.

Regarding the possibility to change the optical absorption of the NPs by the influence of an ultrasonic effect, the modification of the amplitude of near-resonance optical signals in the sample was considered. In Figure 9, the sawtooth waveform corresponds to the excitation of plasmonic signals induced by a 532 nm wavelength exciting the SPR associated with the Au atoms of the samples. The value of the single-beam transmittance measurement with and without the participation of an ultrasonic control was analyzed. In addition to this experiment, an ultrasonic signal was propagated from the transversal direction to the laser beam, which passed through the sample cell containing Au-Pt NPs in ethanol. The data plotted in red color were obtained by modulating the amplitude in the US frequency of 40 kHz. The changes in the y-axis of Figure 9 correspond to the transmittance when an Nd:YVO4 pulsed laser system irradiates the sample. The resulting data were obtained for two different cases; initially, a PIN diode (PD 2) at the output of measured the value when the laser irradiates the sample with a repetition period of T = 1/50 kHz = 0.02 ms. Then, the optical transmittance was measured in simultaneous propagation of the US square wave, delivered at a pulse repetition frequency of 40 kHz over a total exposure time of 5 s.

One interesting observation is the monotonic decrease in the plasmonic and optical transmittance in a linear relationship with the US increase in good agreement with previous studies [43]. Low-level ultrasonic signals were systematically employed with repetitive results within an error bar below 1%. The possibility to change the bimetallic structure exhibited by the NPs under the influence of US waves was considered to be part of further investigations. Change in metallic structures has been previously reported [55] and also the effects of grain size decrease by US waves [56].

All the measurements were carried out in the presence of the US perturbation, also was performed a base data acquirement to observe the influence of the US in the studied Au-Pt NPs. We observed the highest contribution of the US in the optical response of the NPs when the US source was in the neighborhood of the surface of the sample; distances of several centimeters practically inhibit the influence of the US frequency in the optical effects evaluated. The modification of the optical transmittance as a function on US signal can be explained by considering that the US frequency is capable to modify the optical and the refractive index; since both are strongly dependent of the vibrational mode of the sample. The study of NPs and nanofluids are of great interest in nanofluidic electronic circuits to manipulate ions and create biomolecular diagnosis devices [1]. The idea of constructing some new devices based on the combination or rearrangement of diodes is found to be applicable in nanofluidics. *p–n* junction diodes are the elementary building blocks of most semiconductor devices [57]. In the feedback of all-nanoparticle logic systems, can be oppositely charged by using laminated nanoparticle layers. Nanostructured devices based on the advantages of plasmonic effects can be found in bipolar junction transistors, field-effect transistors or thyristors [58]. Doped *p–n* junctions have been created considering electrostatics instead of adding different chemical elements in single configurations [59]. A chemoelectronic circuit composed of Au NPs coated with different types of organic molecules has been recently suggested for the development of diodes/transistors. In this respect, the major interest is manipulating the ions’ movement of the packed Au NPs in an inherent electric field [56].

Particularly, hybrid nanoscale acousto-optic systems have been proposed as acousto-optic sensors, acousto-optic wireless communication modulators and transducers based on graphene [60]. As well, Au NPs have been studied by using acousto-plasmonic interactions [26]. However, the optical and catalytic properties can be improved by combining two different metals at nanometric scale [61], in which, metal composition and shape provide unique functionality [62]. Among the different bimetallic NPs, Au-Pt NPs show higher electrocatalytic activity that represents widely promising functions in catalysis applications, such as a suitable catalyst for methanol oxidation and oxygen reduction reaction [63]. The enhanced photocatalytic activity is attributable to the synergy of the metal composition and the SPR effects.

It has been previously reported that third-order optical properties may conduct to a larger sensitivity to local changes in the refractive index compared to the commonly used linear localized SPR sensing [64]. It has been demonstrated that the optical transmission of nanoplasmonic materials becomes spectrally tunable by acoustic waves [65]. Moreover, monotonic changes in the sensitivity exhibited by nonlinear optical sensors have been indicated [43]. Contrastingly, in this work, we pointed out the possibility to increase or decrease the modulation of optical signals by linear or nonlinear optical effects; respectively, in plasmonic NPs.

The third-order nonlinear optical susceptibilities can be responsible for the change in the refractive index and the nonlinear absorption of colloidal solutions irradiated by light [66]. These changes in refractive index automatically modify the mechanical properties of the sample that depend on mass density. Specifically, light-sound interactions have been used for developing nonlinear acousto-optical modulation which depends on the resonance and the vibrational mode of the metal nanostructures [30]. We noted that the influence of US in nonlinear optical properties can generate a modification in optical transmittance that is opposite to the modification of the propagation of light in the linear regime. Our results are in good agreement with nonlinear optical effects in liquids that demonstrate the possibility to modify the refractive index [67] taking into account that plasmonic resonances show considerable changes in optical properties exhibited by nanocomposites [68]. Comparatively, herein is reported the observation of ultrasonic influence and their potential to separately modulate with opposite behavior the linear and nonlinear optical effects in plasmonic NPs. In this work has been highlighted the important influence of simultaneous interaction of both optical and ultrasonic signals to control plasmonic phenomena.

## 4. Conclusions

A strong influence in the modulation of the optical and plasmonic properties exhibited by bimetallic NPs was demonstrated by using ultrasonic waves. Spectral and TWM experiments in an Au-Pt based nanofluid under the influence of ultrasonic signals were reported. A reduction in the magnitude of the nonlinear optical transmittance dependent on external mechanical perturbations was observed. Herein is identified that the US wave propagation in nanostructured materials can be useful for controlling nonlinear and plasmonic signals. From the point of view of quantum optics, NLO receives an important influence from plasmonics and mechanical effects. Then the study of the modification of nonlinear optical phenomena assisted by US signals promises to be effective in a wide range of applications for instrumentation and low-dimensional signal processing where mechanical and plasmonic actions occur. The ultrasonic and nonlinear optical behavior of bimetallic Au-Pt NPs can be considered as potential candidates to develop quantum operations, sensors and instrumentation devices in biomedicine.
